# Co-existence of Phenylketonuria and Fabry disease on a 3 year-old boy: case report

**DOI:** 10.1186/1471-2431-10-32

**Published:** 2010-05-17

**Authors:** Daniela Concolino, Maria Rapsomaniki, Eliana Disabella, Simona Sestito, Maria G Pascale, Maria T Moricca, Giuseppe Bonapace, Elisea Arbustini, Pietro Strisciuglio

**Affiliations:** 1 Department of Pediatrics, University "Magna Graecia", Catanzaro, Italy; 2 Centre for Inherited Cardiovascular Diseases, I.R.C.C.S. Policlinico San Matteo, Pavia, Italy; 3 Department of Pediatrics, University "Federico II", Naples, Italy

## Abstract

**Background:**

The co-existence of two genetically distinct metabolic disorders in the same patient has rarely been reported. Phenylketonuria (PKU) is an inborn error of the metabolism resulting from a phenylalanine hydroxylase deficiency. Fabry disease (FD) is an X-linked lysosomal storage disorder due to a deficiency of the enzyme alpha-galactosidase A.

**Case presentation:**

We report a case of a 3 year- old boy affected by classic PKU and FD, both confirmed by molecular data. The FD was suspected at the age of 21 months on the presence of non-specific GI symptoms (severe abdominal pain and periodically appearance of not specific episodes of gastroenteritis) apparently non related to PKU.

**Conclusion:**

This is the first report of co-existence of FD and PKU, two different congenital inborn of metabolism and in consideration of the prevalence of each disease this chance association is a very unusual event. The co-existence of this diseases made very difficult the correct interpretation of clinical symptoms as lack of appetite, severe abdominal pain and non-specific gastroenteritis episodes. Furthermore, this case report helps to define the early clinical phenotype of FD.

## Background

The presence of two genetically distinct metabolic diseases in the same patient has rarely reported. Phenylketonuria (PKU) (OMIM 221600) is inborn error of the metabolism resulting from a deficiency of phenylalanine hydroxylase (PAH). The *Phenylalanine (Phe) hydroxylase *gene is located on chromosome 12q22-q24.1 and so far more than 400 different mutant Phe hydroxylase alleles have been described[1]. A Phe restricted diet can improve the effects of high serum Phe on cognitive functions. Fabry disease (FD) (OMIM 301500) is an X-linked lysosomal storage disorder due to a deficiency of the enzyme alpha-galactosidase A (GLA). The *GLA gene *maps at Xq22.1 and more than 400 mutations have been recorded in the Human Gene Mutation Database [2]. The disease affects kidney, myocardium, central nervous system, heart, ears, eyes, skin and, in many patients, the gastrointestinal tract (GI) [3]. FD can become clinically manifest in childhood with chronic neuropathic pain, hypohidrosis/hyperhidrosis, angiokeratoma, acroparaesthesiae, non-specific GI symptoms and hearing problems [4]. Recently, recombinant enzyme replacement therapy (ERT) has become available. The diagnosis of FD is often delayed in the pediatric population. The symptoms may appear in a nonspecific pattern in this age group, and often many years are required to identify the underlying nature of the complaints. Pedigree studies and a careful family history are important and in the absence of such information, the diagnosis of FD, confirmed by enzyme/genetic analysis, is usually suspected on the basis of strong clinical signs and recognition of the peculiarity of the clinical findings [5]. We report a new pediatric case of FD with early GI involvement in a 3- year-old boy affected by PKU.

## Case presentation

**Figure 1:**
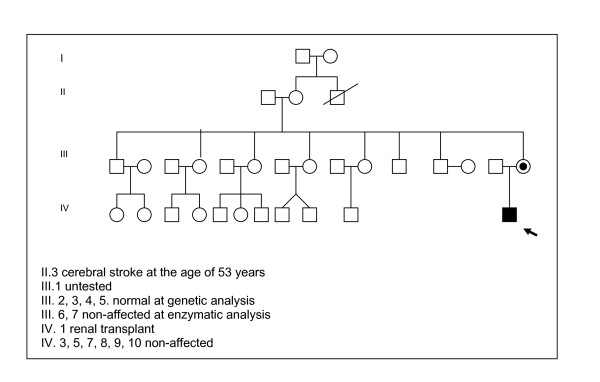
**Family pedigree**.

A 3 -year-old boy was first seen in our department at the age of 20 days for a positive neonatal screening for PKU. He was born at 41 weeks of gestation to non-consanguineous parents, with a birth weight of 2810 g (10th centile). Family history was unremarkable for FD except for a maternal uncle deceased at the age of 53 years of cerebral stroke, and a grandmother suffers of systemic hypertension. A maternal niece has developed severe renal failure at birth and underwent to renal transplantation at the age of 19 years for polycystic kidneys (Fig. 1). In our child an hyperphenylalaninemia due to BH4-deficiency was excluded by BH4 loading test (20 mg/Kg), analysis of urinary pterins, and determination of dihydropterine reductase activity on blood spot. Moreover, mutation analysis by restriction enzyme analysis was used to identify his genotype resulting homozygous for c.143 T>C mutation on exon 2 that changes in the protein Lys with Ser (p.L48S). The patient was subjected to a low-Phe diet and supplementation with Phe-free amino-acid mixtures. Treatment compliance was excellent with a mean of serum Phe value ~3 mg/dl during the day and good tolerance (440 mg Phe/day). Growth, neurological and psychological development were normal. At the age of 28 months, the boy began to show a slow growth, lack of appetite, severe abdominal pain and appearance often of episodes of gastroenteritis, resolving spontaneously and not related with the diet treatment. Laboratory routine investigations, sweat test, acid-base status, inflammatory parameters, stool culture, oven and parassite, faecal occult blood test, antigliadin antibodies (AGA), antiendomisium antibodies (EMA), tissue transglutaminase (tTG), were in the normal range. Symptoms of non specific enteropathy, such recurrent diffuse abdominal pain and diarrhea continued and at the age of 36 months he showed a deceleration of linear growth velocity (from 50^th ^centile to 10-25^th ^centile) although the BMI was in normal range. These symptoms combined with the familiar history, suggested us a possible FD. At clinical examination no angiokeratomas were found.

The diagnosis was confirmed by the reduced plasma a-galactosidase A (0,32 nmol/h/ml), decreased activity assayed in leucocytes [0,65 nmol/mg.prot/h (normal range: 26-80)] and the molecular analysis of *GLA gene *documented the hemizygous missense mutation g.5234G>A 2 in the exon 2 that changes in the protein Arg with Hys (p.R112H). In the mother, the mutation analysis showed that she carries the same heterozygous mutation. We performed a systematic pedigree analysis by the assay of *GAL *activity in the family and mutation analysis to brothers, sisters, nephews of the mother that resulted normal. The grandmother (II.2) and a brother (III.1) of the mother refused to undergo to molecular analysis (Fig. 1).

Due to the lack of other clinical signs we decided not to start ERT but to perform a clinical follow-up every 6 months. Today the patient is 5-year-old, continues a diet therapy for PKU and severe abdominal pain is present periodically without triggering factors in absence of other symptoms.

## Conclusion

The clinical relevance of this case is the combination of two different metabolic inherited diseases and the early presentation of FD. The co-existence of PKU and FD made very difficult the correct interpretation of clinical symptoms as lack of appetite, severe abdominal pain and non-specific gastroenteritis episodes. In fact GI symptoms and feeding problems also in young PKU children are present [6]. Non specific enteropathy has been reported in about 80% of pediatric FD patients^4 ^and these symptoms must alert pediatricians to possible FD, also because the incidence of the disease seems higher than previously estimated [7]. It is, therefore, difficult predicting the clinical outcome in this young patient who is still free of organ involvement. The mutation p.R112 H found in our patient has been previously identified in patients with either the classic phenotype or the cardiac-variant phenotype [8]. Given that the reversibility of disease progression in adulthood is limited, early ERT appears reasonable, because the disease progresses with age. However, the optimum time to start ERT in order to prevent end-stage organ damage is also unknown. It is possible that beginning of therapy at an early age, before the occurrence of significant organ damage and dysfunction, produces better clinical results. Moreover, little is known about the natural history of the disease in childhood, and systematic prospective data on the clinical manifestations of FD by ERT in the pediatric population are not available. It is essential, however, to study the therapeutic and possibly preventive effects of this treatment approach on children in a controlled way. Clinical trials are currently underway to evaluate the safety and efficacy of enzyme replacement therapy in children with FD [9]. According to current expert recommendations, ERT in male patients should ideally be provided in the second decade of life or as soon as clinical signs and symptoms, such as left ventricular hypertrophy, proteinuria, hypoidrosis or pain, are observed [8]. Further, based on our knowledge, cases of FD and PKU have been never described in the same patient. Finally, this case emphasizes the need to look for the coexistence of other inherited metabolic diseases when the clinical history is not completely consistent with the well assessed first diagnosis.

## Consent

Written informed consent was obtained from the parents of the patient for publication of this case report and accompanying images. A copy of the written consent is available for review by the Editor-in-Chief of this journal.

## Competing interests

The authors declare that they have no competing interests.

## Authors' contributions

DC, MR and SS wrote the case report. MGP supervised the therapy. ED and EA made the molecular analysis. MTM and GB made the biochemical analyses. PS edited the report. All authors read and approved the final manuscript.

## Pre-publication history

The pre-publication history for this paper can be accessed here:

http://www.biomedcentral.com/1471-2431/10/32/prepub
